# Prion protein is required for tumor necrosis factor α (TNFα)-triggered nuclear factor κB (NF-κB) signaling and cytokine production

**DOI:** 10.1074/jbc.M117.787283

**Published:** 2017-09-12

**Authors:** Gui-Ru Wu, Tian-Chen Mu, Zhen-Xing Gao, Jun Wang, Man-Sun Sy, Chao-Yang Li

**Affiliations:** From the ‡ Wuhan Institute of Virology, Chinese Academy of Sciences, State Key Laboratory of Virology, 44 Xiao Hong Shan Zhong Qu, Wuhan 430071, China,; the § University of Chinese Academy of Sciences, Beijing 100000, China; the ¶ Department of Life Sciences, Wuhan University, Wuhan 430010, China,; the ‖ Department of Pathology, Case Western Reserve University, Cleveland, Ohio 44106, and; the ** Wuhan Brain Hospital, No. 5 Huiji Road, Jiang'an District, Wuhan 430010, China

**Keywords:** deubiquitylation (deubiquitination), melanoma, NF-κB (NF-κB), prion, tumor necrosis factor (TNF), CYLD, NF-κB signaling, PrP, TNFα

## Abstract

The expression of normal cellular prion protein (PrP) is required for the pathogenesis of prion diseases. However, the physiological functions of PrP remain ambiguous. Here, we identified PrP as being critical for tumor necrosis factor (TNF) α-triggered signaling in a human melanoma cell line, M2, and a pancreatic ductal cell adenocarcinoma cell line, BxPC-3. In M2 cells, TNFα up-regulates the expression of p-IκB-kinase α/β (p-IKKα/β), p-p65, and p-JNK, but down-regulates the IκBα protein, all of which are downstream signaling intermediates in the TNF receptor signaling cascade. When *PRNP* is deleted in M2 cells, the effects of TNFα are no longer detectable. More importantly, p-p65 and p-JNK responses are restored when *PRNP* is reintroduced into the *PRNP* null cells. TNFα also activates NF-κB and increases TNFα production in wild-type M2 cells, but not in PrP-null M2 cells. Similar results are obtained in the BxPC-3 cells. Moreover, TNFα activation of NF-κB requires ubiquitination of receptor-interacting serine/threonine kinase 1 (RIP1) and TNF receptor–associated factor 2 (TRAF2). TNFα treatment increases the binding between PrP and the deubiquitinase tumor suppressor cylindromatosis (CYLD), in these treated cells, binding of CYLD to RIP1 and TRAF2 is reduced. We conclude that PrP traps CYLD, preventing it from binding and deubiquitinating RIP1 and TRAF2. Our findings reveal that PrP enhances the responses to TNFα, promoting proinflammatory cytokine production, which may contribute to inflammation and tumorigenesis.

## Introduction

The normal cellular prion protein (PrP)^2^ is a widely expressed, highly conserved glycosylphosphatidylinositol (GPI) anchored, cell-surface glycoprotein (1, 2). Despite extensive studies, the normal physiologic functions of PrP remain an enigma (1, 2). Genetically engineered mice without a functional *Prnp* are viable, reproduce normally, and exhibit no discernable pathological phenotypes (3, 4). Goats naturally born without a functional *Prnp* due to a stop-codon mutation are also normal (5). True heterozygous loss-of-function alleles of *PRNP* are found in apparently healthy humans (6). Nonetheless, more than 50 ligands have been reported to bind PrP. These ligands include cell-surface proteins, cytoplasmic proteins, nucleic acids, divalent cations, lipids, and glycosaminoglycans (7–16). PrP is detected on the cell surface, in the cytoplasm, mitochondria, and nucleus (17–28). Interactions between PrP and these ligands participate in a plethora of biological responses, such as apoptosis, cell adhesion, migration, proliferation, pro-inflammatory cytokine production, metal homeostasis, signal transduction, and regulation of transcription (16, 26–33). Hence, the roles PrP play in these responses are clearly cell-context dependent.

PrP is expressed in some but not all lymphoid cells (34). PrP modulates T cell activation (35). PrP on the cell surface is released upon activation (36, 37). Although PrP is not required for mast cell differentiation, it is released *in vivo* in responding to allergens (38). In normal skin, a low level of PrP is detected mostly in keratinocytes (39). However, in inflammatory skin diseases, such as psoriasis and contact dermatitis, PrP was up-regulated in keratinocytes and infiltrating mononuclear cells (39). In monocytes IFN-γ modulates the expression of PrP (40). PrP also regulates phagocytic activity and inflammatory responses of macrophages (41, 42). After dextran sodium sulfate treatment, PrP null mice expressed higher levels of proinflammatory cytokines, such as IL-1β, IL-6, TNFα, IL-4, IFN-γ, and BAD compared with wild-type mice (43, 44). PrP was essential for the protection of mice when challenged with LPS (45). Collectively, these findings suggest that PrP plays critical roles in modulating inflammatory responses.

Persistent NF-κB activation has been reported in several human cancers (46). Up-regulation of PrP has also been reported in cancers (47–50). However, the underlying mechanisms by which PrP promotes tumor growth are not completely understood.

Previously, we reported that in some human PDAC cell lines, such as BxPC-3 and a melanoma cell line, M2, PrP exists as pro-PrP as defined by retaining its GPI-peptide signaling sequence (47, 51). The GPI-peptide signaling sequence of PrP contains a filamin A (FLNa) binding motif and thus, binds FLNa. FLNa is a cytolinker protein that links cell-surface receptors to the cytoskeleton (52, 53). Binding of pro-PrP to FLNa disrupts the normal physiologic function of FLNa and renders the tumor cells more aggressive and invasive *in vitro* and *in vivo*. Most importantly, expression of PrP is a marker of poorer prognosis in patients with PDAC (47).

Melanoma cell line, M2, expresses pro-PrP but lacks FLNa (48). To study whether PrP has additional functions independent of binding FLNa, we used the CRISPR/Cas approach (54) to delete *PRNP* in M2 and BxPC-3 cells. We then compared the biological discrepancies of wild-type M2 and BxPC-3 cells with their corresponding PrP null cells. We found that expression of PrP is required for TNFα-triggered NF-κB signaling and TNFα production in these cells. Therefore, in addition to binding FLNa, PrP may promote inflammation, contributing to tumor growth and progression.

## Results

### PrP is required for responses to TNF receptor signaling in M2 cells

**Figure 1.**
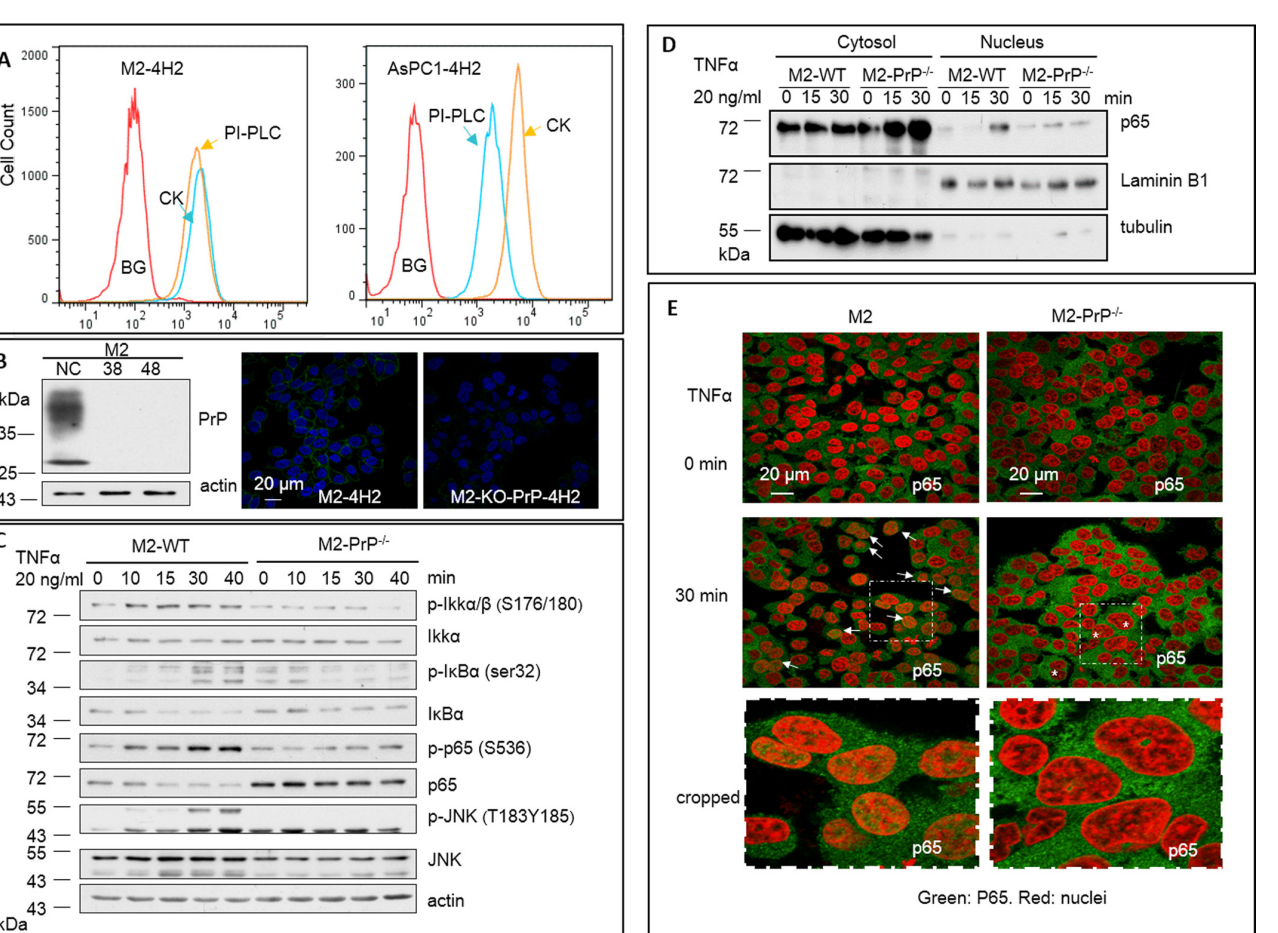
**PrP is required for responses to TNFα signaling in human melanoma cell line M2.** *A,* melanoma M2 cells expressing PI-PLC resistant PrP on the cell surface. *BG*, background, M2 cells without PI-PLC treatment were stained with IgG1 isotype control. *PI-PLC*, M2 cells treated with PI-PLC were stained with 4H2. *CK*, M2 cells treated with control vehicle were stained with 4H2. *B*, immunoblotting confirmed that knock-out of *PRNP* in M2 cells. *38* and *48* were two *PRNP* null M2 cell lines. Confocal immunofluorescence staining with 4H2 further confirmed that *PRNP* was deleted in M2 cells. M2 cells showed a positive 4H2 staining signal (*left panel*), whereas M2 *PRNP* null M2 cells showed negative PrP staining (*right panel*). *C,* M2 wild-type cells but not M2-PrP^−/−^ cells showed obvious activation of NF-κB signaling. Sustained activation of p-Ikkα/β (*S176/180*), p-p65 (*S536*), p-IκBα (*ser32*), and p-JNK were detected in M2 cells after stimulation with TNFα compared with M2-PrP^−/−^ cells. *D,* treatment with TNFα resulted in p65 translocated into nucleus in M2 cells. A significant amount of nuclei p65 was detected in M2 cells but not *PRNP* null M2 cells 30 min after TNFα treatment. *E,* confocal immunofluorescence staining of p65 showed nuclei p65 staining in M2 cells treated with TNFα. Very few nuclei p65 staining was observed in *PRNP* null M2-PrP^−/−^ cells. Enlargement of cropped areas of the pictures were shown. All experiments were repeated three times with similar results.

We stained M2 cells with 4H2, a monoclonal antibody (mAb) specific for PrP, and analyzed the results by flow cytometry. We found that M2 cells indeed expressed PrP on the cell surface, and the PrP was resistant to phosphatidylinositol-specific phospholipase (PI-PLC) treatment (Fig. 1*A*, *top left panel*). Therefore, the PrP in M2 cells was not GPI-anchored and thus a pro-PrP. As expected, PrP on the surface of AsPC-1, a PDAC cell line, known to express a GPI-anchored PrP was sensitive to PI-PLC treatment (Fig. 1*A*, *top right panel*). These results were consistent with our previous findings that the PrP expressed in M2 cells was pro-PrP (48).

To investigate the function of PrP in M2 cells, we used the CRISPR/Cas to delete *PRNP* in M2 cells. Immunoblotting with mAb 4H2 showed that PrP expression was abolished in the two *PRNP* null M2 cell lines, 38 and 48 (Fig. 1*B*, *left panel, 38* and *48*). Confocal immunofluorescence staining for PrP provided additional evidences that PrP was undetectable in these cell lines (Fig. 1*B*, *right panel*). Sequencing of genomic DNA from these cell lines further confirmed that *PRNP* was successfully removed in these two cell lines (results not shown).

Because PrP has been implicated in inflammation response, we then compared PrP^+/+^ M2 cells with its PrP null derivatives for their response to a panel of mediators, such as TNFα, IL-1β, EGF, or a toll-like receptor (TLR) agonist. The signaling cascades of these molecules share many common intermediates, such as up-regulation of p-IKKα/β and p-p65 and down-regulation of IκBα, eventually leading to activation of NF-κB, translocation of NF-κB complex into the nucleus, and transcription of the NF-κB responsive genes. Thus, we used these markers to assess whether PrP was required for M2 cells to respond to these exogenous ligands (55, 56). We found that only TNFα but not EGF, IL-1β, and LPS stimulated the phosphorylation of the p-IKKα/β complex in PrP^+/+^ M2 cells, in a time-dependent manner (Fig. 1*C* and supplemental Fig. S1). The response was first detected at 10 min and peaked at 40 min. Interestingly, up-regulation of p-IKKα/β was undetectable in PrP null M2 cells. Identical treatment did not significantly alter the level of total IKKα (Fig. 1*C*). In the TNFR1 signaling cascade, IκBα is phosphorylated by IKKs (57). Thus, IκBα should respond similarly to IKKα/β. As expected, an obvious up-regulation of p-IκBα was detected at 10 min after TNFα treatment and persisted for at least 40 min in the M2 wild-type cells (Fig. 1*C*). Upon activation, IκBα is ubiquitinated and degraded (58). Accordingly, we also detected an obvious reduction in the level of total IκBα (Fig. 1*C*). However, in PrP null M2 cells, TNFα treatment barely altered the levels of either p-IκBα or total IκBα (Fig. 1*C*).

Activation of p65, a subunit of NF-κB may result in its phosphorylation and proteasome-dependent degradation (59–62). Thus, we investigated whether p65 was activated in TNFα-treated M2 wild-type cells. We found that after treatment with TNFα for 10 min, there was a significant elevation in the level of p-p65 (Fig. 1*C*). We also observed obvious p65 reduction, starting at 15 min after TNFα treatment (Fig. 1*C*). On the contrary, TNFα treatment of PrP null M2 cells did not show any increase in p-p65, and there was also no significant decrease in the level of total p65 (Fig. 1*C*). On the contrary, due to augmented transcription (supplemental Fig. S2), the basal level of p65 is higher in PrP null M2 cells (Fig. 1*C*).

TNFα activation causes the phosphorylation and activation of JNK (63). We found that when treated with TNFα, there was a higher level of p-JNK (Thr^183^ and Tyr^185^) in PrP^+/+^ M2 cells but not in PrP null M2 cells (Fig. 1*C*). TNFα treatment did not affect the level of total JNK in PrP^+/+^ M2 cells but curtailed the level of total JNK in PrP null M2 cells (Fig. 1*C*).

### Translocation of p65 in M2 cells is dependent on PrP

Activated p65 forms heterodimer with p50 or p52, the complex is then translocated to the nucleus to initiate transcription (64). Hence, we investigated whether p65 is translocated to the nuclei after TNFα stimulation. We found that significantly more p65 were detected in the nuclei fraction after treatment with TNFα (Fig. 1*D*) in M2 cells. In contrast, in PrP null M2 cells, TNFα treatment did not increase the level of p65 in their nuclei fraction (Fig. 1*D*). Immunoblotting for tubulin and laminin B1 in respective fractions confirmed the efficacy of the fractionation (Fig. 1*D*).

To seek additional support for our conclusion that p65 enters the nucleus after TNFα stimulation, we performed confocal immunofluorescence staining for p65. We found that in PrP^+/+^ M2 cells, there were more p65 signals in their nuclei after TNFα treatment compared with untreated M2 cells (Fig. 1*E*, indicated by *arrows, green* color is p65 staining; *red* color is nuclei staining). On the contrary, TNFα stimulation did not enhance the translocation of p65 into the nuclei in PrP null M2 cells (Fig. 1*E*). These results provide strong evidence that expression of PrP is critical for responding to TNFα-triggered NF-κB activation in M2 cells.

### Response to TNFα treatment is rescued when PRNP is re-introduced into PrP null cells

**Figure 2.**
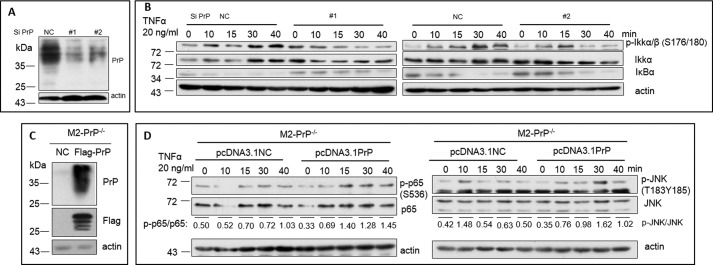
**Response to TNFα treatment is rescued when *PRNP* is re-introduced into PrP null cells.** *A,* down-regulation of PrP with two pairs of siRNA oligos was confirmed by immunoblotting with PrP specific mAb 4H2. *NC*, negative control siRNA, #*1* and #*2* represent two different target sequences. *B,* down-regulation of PrP with siRNA reduced p-IKKα/β (*S176/180*) and IκBα after TNFα treatment. *C,* immunoblotting showed PrP expression in rescued cells (*FLAG-PrP*) but not in control cells (*NC*). *D,* comparing to control *PRNP* null M2-PrP^−/−^ cells, PrP rescued M2 cells showed a time dependent up-regulation of p-p65 (*S536*) and p-JNK (*T183/Y185*) upon TNFα treatment, respectively. All experiments were repeated three times with similar results.

To eliminate the possibility that PrP null M2 cells did not respond to TNFα was due to an off-target effect of CRISPR/Cas, first, we performed PrP down-regulation with siRNA and found that down-regulation of PrP (Fig. 2*A*) did impact M2 cells response to TNFα (Fig. 2*B*). On the other hand, we re-introduced a FLAG-tagged PrP back into PrP null M2 cells. Anti-FLAG-specific antibody and anti-PrP Mab (4H2) identified PrP expression in the *PRNP* rescued cells but not in the control cells (Fig. 2*C*). We then stimulated the PrP rescued cells and irrelevant rescued control PrP null cells with TNFα. We found that p-p65 was significantly up-regulated in *PRNP* rescued cells but not in the control cells (Fig. 2*C*). In addition, we detected reduced basal level of p65 in *PRNP-*rescued cells (Fig. 2*D*). This result, together with decreased p65 in PrP negative cells (Fig. 1*C*), suggest that there is a functional interplay between PrP and p65. Moreover, we also detected an up-regulation of p-JNK in *PRNP*-rescued cells (Fig. 2*D*). Unfortunately, the expression levels of p-IKKα/β, p-IκBα, and JNK could not be rescued even when *PRNP* was re-introduced into *PRNP* null M2 cells (data not shown). The exact reason for this discrepancy is not clear. It is possible that the levels of these molecules are regulated by other molecules in addition to PrP, or one or more of these molecules could be altered due to the off-target effects of CRISPR/Cas or PrP overexpression. Nonetheless, these results support our conclusion that the lack of p65 activation observed in PrP null M2 cells is most likely due to elimination of *PRNP*.

### PrP is required for the transcriptional activity of NF-κB and production of TNFα

**Figure 3.**
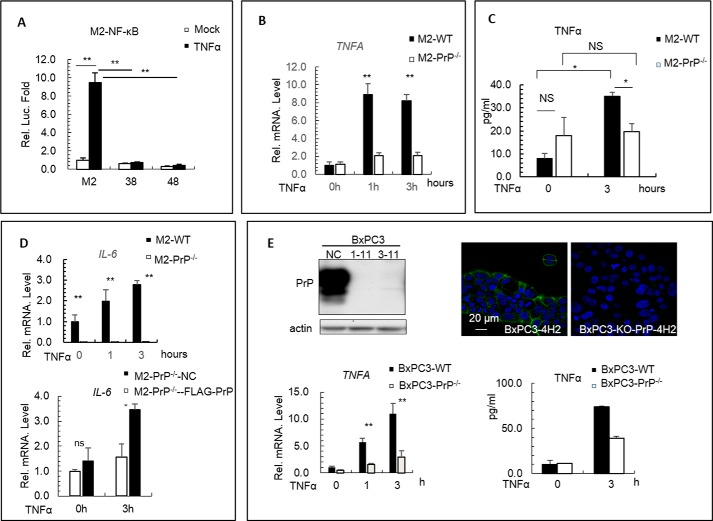
**PrP is required for the transcriptional activity of NF-κB and production of TNFα.** *A,* in the presence of PrP, treatment of M2 cells with TNFα (*black box*) significantly induced the NF-κB reporter activity compare with untreated M2 cells (*p* < 0.01). On the contrary, treatment of *PRNP* null M2-PrP^−/−^ cells (*38* and *48*) did not induce the activity NF-κB reporter (*p* < 0.01). *B,* treatment of M2 cells (*black box*) with TNFα significantly stimulated *TNFA* mRNA synthesis. On the contrary, treatment of *PRNP* null M2-PrP^−/−^ cells (*open box*) did not activate *TNFA* mRNA synthesis. *C,* treatment of M2 cells with TNFα (*black box*) significantly stimulated TNFα production in the culture medium compared with that of untreated cells (*p* < 0.05). On the contrary, treatment of *PRNP* null M2-PrP^−/−^ cells (*open box*, mixture of 38 and 48) did not stimulate TNFα production in the culture medium. There was no significant difference (*NS*) of culture medium TNFα level before TNFα treatment comparing M2 cells with M2-PrP^−/−^ cells. However, there was a significant difference in the TNFα level 3 h after TNFα treatment comparing M2 cells with M2-PrP^−/−^ cells (*p* < 0.05). *D,* treatment of M2 cells (*top panel*, *black box*) with TNFα stimulated *IL-6* mRNA synthesis. On the contrary, treatment of *PRNP* null M2 (*top panel*, *open box*) did not activate *IL-6* mRNA synthesis. *E,* loss of PrP in BxPC-3 cells inhibited the production of TNFα. Immunoblotting confirmed knock-out of *PRNP* in BxPC-3 cells. *1–11* and *3–11* were two *PRNP* null BxPC-3 cell lines (*top left panel*). Confocal immunofluorescence staining with 4H2 confirmed that *PRNP* was deleted in *PRNP* null BxPC-3 cells (*top right panel*). TNFα treatment induced cytokine expression in BxPC-3 cells. Treatment of BxPC-3 cells (*black box*, *bottom left panel*) with TNFα stimulated *TNFA* mRNA synthesis compared with the *PRNP* null BxPC-3-PrP^−/−^ cells (*open box*, *bottom left panel*). TNFα treatment induced the production of the culture medium TNFα level after 3 h compared with *PRNP* null BxPC-3 cells (*bottom right panel*). All experiments were repeated three times with similar results except the induction of TNFα level in BxPC3 and its *PRNP* null cells.

Next, we used the NF-κB reporter assay to investigate whether PrP was required for NF-κB transcriptional activity. PrP^+/+^ M2 cells and PrP null cells were transfected with either a control plasmid or a NF-κB reporter plasmid. Transfected cells were then stimulated with TNFα. We found that in contrast to PrP^+/+^ M2 cells, there was little NF-κB transcriptional activity in PrP null M2 cells (Fig. 3*A*). Thus, PrP is required for the transcriptional activity of NF-κB. NF-κB activates the production of pro-inflammatory cytokines, such as TNFα and IL-6. Next, we investigated whether TNFα induces TNFα and other cytokine production in M2 cells. First, we measured TNFα mRNA at different time points after TNFα stimulation. We found that treatment with TNFα significantly stimulated the production of TNFα mRNA in PrP^+/+^ M2 cells in a time-dependent manner (Fig. 3*B*, *solid columns*). However, the responses were significantly reduced in PrP null M2 cells (Fig. 3*B*, *open columns*). Next, we used enzyme-linked immunosorbent assays (ELISA) to confirm that TNFα indeed stimulated more TNFα in PrP^+/+^ M2 cells than PrP null cells. TNFα treatment increased the level of TNFα from 8 to 32 pg/ml in the culture supernatant of M2 cells (Fig. 3*C*, *left panel*, *solid columns*). This increase was not observed in TNFα-treated PrP null M2 cells (Fig. 3*C*, *left panel*, *open columns*). In addition to *TNFA*, we found that *IL-6* mRNA was also significantly up-regulated in a time-dependent manner in M2 cells (Fig. 3*D*, *solid columns*, *top panel*) but not in PrP null M2 cells (Fig. 3*D*, *open columns*, *top panel*). We then checked *IL-6* mRNA in PrP-rescued M2 cells. We found that when treated with TNFα, PrP rescued M2 cells synthesized significantly more *IL-6* mRNA than control cells (Fig. 3*D*). Thus, there is a functional interplay between PrP expression and IL-6 transcription under TNFα treatment. However, the underlying mechanism linking these two events remains to be investigated. On the other hand, We did not detect up-regulation of *IL-8* mRNA in PrP positive M2 cells under TNFα treatment (results not shown).

To seek additional support for our observation on the functional interplays between PrP and NF-κB activation, we carried out similar studies in a PrP^+/+^ PDAC cell line, BxPC-3. Similar to M2 cells, the PrP expressed in BxPC-3 is also pro-PrP (47, 51). When the *PRNP* was deleted in this cell line (Fig. 3*E*, *top panel*), they also showed significant reduction in the level of *TNFA* mRNA when stimulated with TNFα (Fig. 3*E*, *bottom panel*). The target sequence used to delete *PRNP* in BxPC-3 cell was distinct from the one used to eliminate *PRNP* in M2 cells (Table 1). This result further reduced the possibility that the reduction of NF-κB activation observed in PrP null M2 cells and in PrP null BxPC-3 cells was due to an off-target effect of CRISPR/Cas.

**Table 1:** **Primers used to generate *PRNP* null M2 and BxPC-3 cells**

Primer name	Sequence
KO PrP in M2	
Sense	5′-CACCGGTGGTGGCTGGGGTCAAGG-3′
Antisense	5′-AAACCCTTGACCCCAGCCACCACC-3′
KO PrP in BxPC-3	
Sense	5′-ACCGGGCTGCCCTGCCCCGGGTAT-3′
Antisense	5′-AAACATACCCGGGGCAGGGCAGCC-3′
Screening F	5′-ATGGCGAACCTTGGCTGCT-3′

### PrP interacts with CYLD to regulate NF-κB signaling

**Figure 4.**
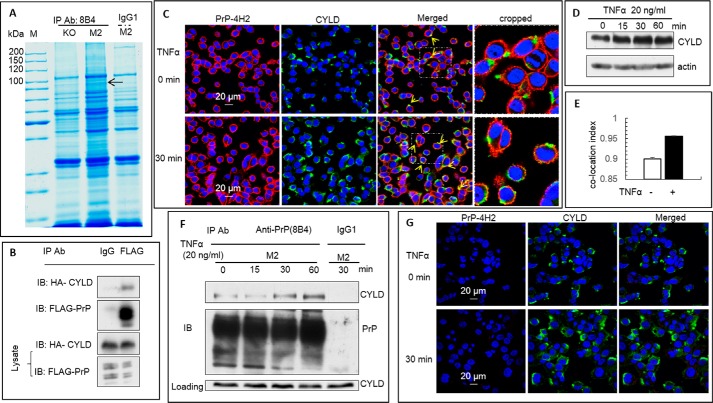
**PrP bound CYLD to regulate NF-κB signaling.** *A,* Coomassie Brilliant Blue staining of proteins co-immunoprecipitated with PrP. *M,* molecular weight marker. *KO, PRNP* null M2 cells. The CYLD corresponding band was labeled with an *arrow. B,* interaction between PrP and CYLD was confirmed in HEK293T cells. Overexpressed FLAG-tagged PrP can immunoprecipitate the HA-tagged CYLD. Equal loading for co-IP was confirmed by detecting the HA-CYLD and FLAG-PrP. *C,* co-location of PrP (*red*) and CYLD (*green*) in M2 cells was increased after TNFα treatment. Co-location was observed around the cellular membrane as indicated by the *arrow*. Increased co-location of CYLD and PrP was detected after 30 min of TNFα treatment (indicated by *arrow*). *Insets* show enlargement of cropped pictures to show more details of the co-location of PrP and CYLD. The elevated CYLD level was also detected. *D,* immunoblotting confirmed that CYLD is up-regulated after TNFα treatment. *E,* statistical analysis of about 200 cells confirmed that more co-location of PrP and CYLD happened after TNFα treatment. *F,* TNFα treatment increased the binding of PrP to CYLD. More CYLD was co-purified with PrP. Equal amounts of loading for CYLD and PrP was confirmed by immunoblotting. *G*, elevation and accumulation of CYLD was PrP independent. All experiments were repeated three times with similar results.

To identify cellular proteins that might interact with PrP to influence the NF-κB signaling in M2 cells, we performed co-IP experiment with another PrP-specific mAb, 8B4. We identified several proteins that were co-purified with PrP in M2 cells but not in PrP null M2 cells (Fig. 4*A*). These protein bands were excised and sequenced by mass spectrometry. Several of these proteins were related to different functions (Table 2). Among these proteins is the tumor suppressor cylindromatosis (CYLD), a de-ubiquitinase known to negatively regulate NF-κB signaling (65). Because CYLD is involved in NF-κB cascade and PrP expression is critical for activation of NF-κB pathway, we thus focused on studying the interaction between PrP and CYLD.

**Table 2:**
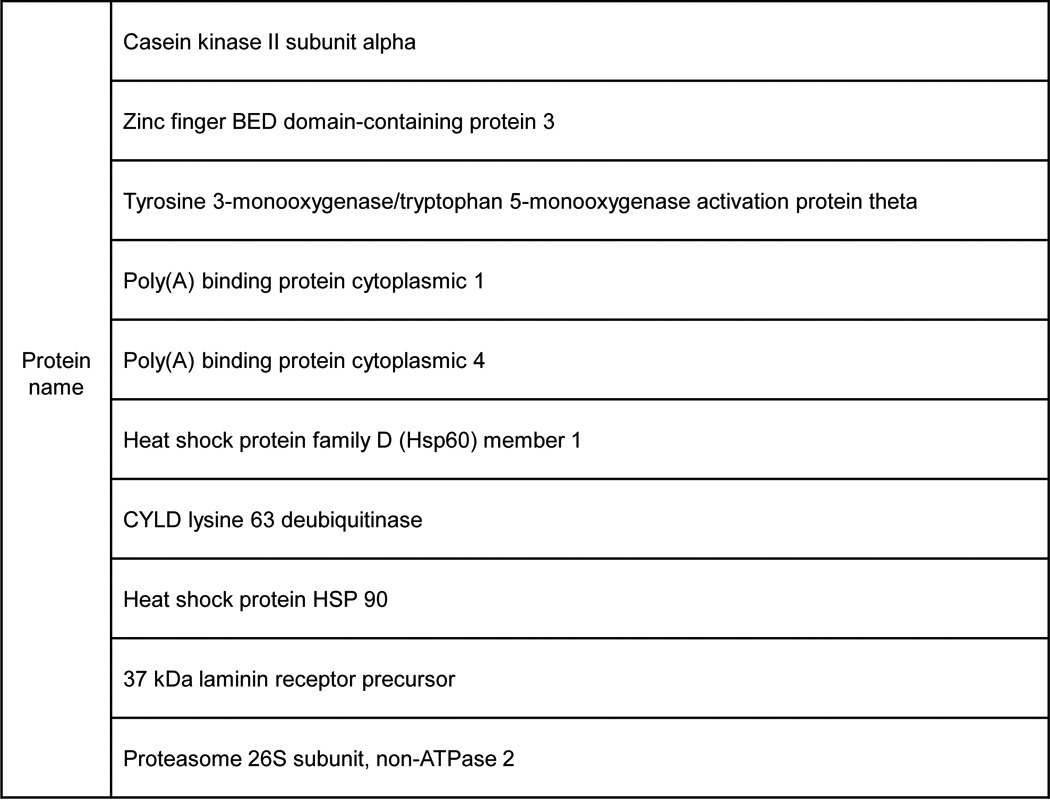
**Proteins identified in the MS results**

To confirm the co-IP results, we expressed a FLAG-tagged PrP and a HA-tagged CYLD in HEK293T cells. In transfected HEK293T cells, CYLD indeed is co-purified with PrP (Fig. 4*B*). Confocal immunofluorescence staining for PrP and CYLD also revealed the co-localization of PrP and CYLD, just underneath the cell surface (Fig. 4*C*, *top panel*). Next, we investigated the physiological consequence of such an interaction after TNFα treatment in M2 cells. We found that treatment with TNFα resulted in a higher CYLD level and more CYLD accumulation on the surface of the M2 cell, which appeared to co-localize with PrP (Fig. 4, *C* and *D*). Quantification of co-localization between PrP and CYLD confirmed that more PrP were co-localized with CYLD after TNFα treatment (Fig. 4*E*). Accordingly, more CYLD was also co-purified with PrP in a time-dependent manner after TNFα treatment (Fig. 4*F*).

To investigate if CYLD up-regulation and accumulation depends on the expression of PrP, we performed confocal immunofluorescence staining of CYLD with *PRNP* null M2 cells before and after TNFα treatment. Similar to PrP^+/+^ M2 cells, up-regulation of CYLD was also observed in *PRNP* null M2 cells after TNFα stimulation (Fig. 4*G*). Thus, TNFα-induced up-regulation and accumulation of CYLD can occur independent of PrP.

### Binding between PrP and CYLD reduces RIP1 and TRAF2 ubiquitination

**Figure 5.**
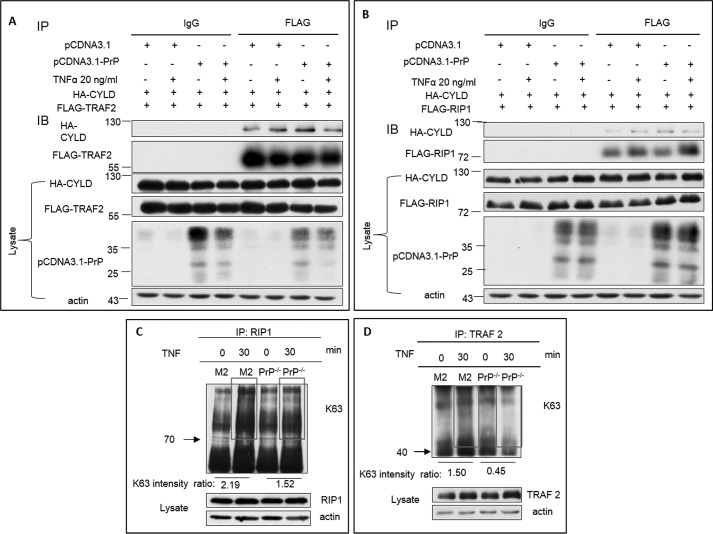
**Interaction between PrP and CYLD increased the ubiquitination of RIP1 and TRAF2.** *A,* reduced binding between CYLD and TRAF2 was detected after TNFα treatment. In the absence of PrP, TNFα treatment slightly increased binding between CYLD and TRAF2 (*top panel*). In contrast, in the presence of PrP, TNFα treatment significantly reduced binding between CYLD and TRAF2 (*top panel*). Loading of CYLD, TRAF2, PrP, and actin were detected with corresponding antibodies (*bottom 4 panels*). Amount of TRAF2 pulled down by antibodies were shown (*second panel*). *B,* reduced binding between CYLD and RIP1 was detected after 30 min of TNFα treatment (*top panel*). Amount of RIP1 pulled down by antibodies were shown (*second panel*). Loading of CYLD, RIP1, PrP, and actin were detected with corresponding antibodies (*bottom 4 panels*). *C,* in PrP expressing M2 cells, TNFα treatment increased RIP1 polyubiquitination, whereas in PrP null M2 cells, TNFα treatment did not increase RIP1 polyubiquitination). (The ratio described the immunoblot intensity in the defined area of TNFα treatment and the untreated as determined by Image J.) *D,* in PrP expressing M2 cells, TNFα treatment increased TRAF2 polyubiquitination, whereas in PrP null M2 cells, TNFα treatment did not increase TRAF2 polyubiquitination. (The ratio described the immunoblot intensity in the defined area of TNFα treatment and the untreated as determined by Image J.)

Activation of NF-κB pathway by TNFα requires K63 polyubiquitination of RIP1 and TRAF2 (66–71). When PrP binds CYLD, it may sequester CYLD, consequentially reducing the levels of CYLD available to bind RIP1 or TRAF2. To test this hypothesis, we expressed HA-tagged CYLD in HEK293T cells, which normally express very low levels of PrP (72). We then transfected FLAG-tagged RIP1 or FLAG-tagged TRAF2 and PrP into these cells. Next, we stimulated the transfected cells with TNFα and performed co-IP experiments. We found that in the absence of PrP, binding between CYLD and TRAF2 was slightly enhanced by TNFα treatment (Fig. 5*A*). On the contrary, in the presence of PrP, binding between CYLD and TRAF2 was reduced (Fig. 5*A*). Similar results were observed between CYLD and RIP1 (Fig. 5*B*). Interactions between CYLD and RIP1 or TRAF2 were specific as isotype controls did not pull down any CYLD (Fig. 5, *A* and *B*). In contrast, under TNFα treatment, binding between CYLD and RIP1 or TRAF2 was reduced, if the cells also expressed PrP (Fig. 5*B*). Thus, in the presence of PrP, TNFα treatment increased the interaction between PrP and CYLD but alleviated binding between CYLD and RIP1 or TRAF2. Because CYLD is a deubiquitinase, reduced binding between CYLD and RIP1 or TRAF2 shall increase the levels of ubiquitination of RIP1 and TRAF2. To verify this possibility, we purified RIP1 and TRAF2 with specific antibodies from M2 cells either treated or untreated with TNFα. The purified proteins were then blotted with K63-specificic polyubiquitin-specific antibody. We found that the levels of polyubiquitinated RIP1 and TRAF2 were significantly increased in the presence of PrP after TNFα treatment (Fig. 5, *C* and *D*). On the contrary, in the absence of PrP, TNFα stimulation did not increase the levels of ubiquitinated RIP1 or TRAF2 (Fig. 5, *C* and *D*).

## Discussion

PrP is up-regulated in many cell types upon cellular activation or viral infection (73–75). However, the physiological consequences of PrP up-regulation remain incompletely understood. We found that when M2 cells were stimulated with TNFα, the TNFR signaling cascade was activated, leading to NF-κB activation and TNFα production. TNFα up-regulates the expression of p-IKKα/β, p-p65, and p-JNK, but reduces the level of IκBα. We provide complementary evidence indicating that PrP is an integrate component of this signaling pathway. When *PRNP* is deleted in M2 cells, these signaling events are greatly reduced. Most importantly, re-introduction of *PRNP* into PrP null M2 cells restores the p-p65 signaling cascade, albeit not as robust as observed in the wild-type M2 cells. Similar results were observed in a PDAC cell line, BxPC-3. When PrP is eliminated from BxPC-3, it also greatly reduced NF-κB activation upon TNFα treatment. Therefore, our observation that PrP is important in TNFα signaling is not limited to M2. Expression of PrP also appears to be important in regulating IL-6 production. However, elucidating the underlying mechanism by which PrP affects the level of IL-6 will require additional studies.

Activation of NF-κB pathway by TNFα requires ubiquitination of RIP1 and TRAF2, which are clients of CYLD (76). We found that in PrP^+/+^ M2 cells PrP is physically associated with CYLD. We posit that when PrP binds CYLD, it sequesters CYLD, preventing it from binding RIP1 and TRAF2. Consequentially, allowing more ubiquitinated RIP1 and TRAF2 to accumulate, and thus promoting NF-κB activation (Fig. 5, *A* and *B*). In the absence of PrP, CYLD is free to de-ubiquintnate RIP1 and TRAF2, reducing NF-κB activation. Interestingly, treatment with TNFα also enhances the CYLD level in PrP null M2 cells. Hence, up-regulation of CYLD by TNFα can occur independent of PrP. Up-regulated CYLD may also contribute to a reduction in NF-κB activation. At this time, the underlying mechanism by which TNFα up-regulates the expression of CYLD is not clear.

CYLD contains three cytoskeletal-associated protein-glycine-conserved (CAP-GLY) domains (77). cytoskeletal-associated protein-glycine-conserved domains are involved in the organization of microtubules and transportation of vesicles and organelles along the cytoskeletal network (78). These processes are important in signal transduction. The PrP in M2 and BxPC-3 cells is a pro-PrP, which interacts with FLNa, a cytolinker protein that is important in organizing actin filaments. Therefore, CYLD and PrP potentially could also mediate their biological effects by modulating the cytoskeletal network. However, FLNa is only present in BxPC-3 cells but not in M2 cells. Therefore, it is unlikely that FLNa will be involved in this pathway.

In normal cells, the PrP is GPI anchored. Thus, it warrants further investigation whether cells expressing a GPI-anchored PrP will respond to TNFα in a manner similar to M2 and BxPC-3 cells. Experiments are also in progress to determine whether the binding between PrP and CYLD is direct. If it is direct, we will determine which domain(s) on PrP and CYLD is/are involved in this interaction. Identifying the domains or the intermediate protein will provide additional insights into the nature of this interaction. If it is indirect, we will identify the intermediate protein by co-immunoprecipitation and mass spectrometry.

Sphingosine 1-phosphate (S1P) is a signaling sphingolipid (79). Binding of S1P to SIPR1 leads to activation of NF-κB (79). It has recently been reported that S1P only activates NF-κB in M2 cells but not in FLNa bearing A7 cells suggesting that FLNa is a negative regulator of NF-κB activation (80). In the same study, it was reported that although TNFα (10 ng/ml) induced robust IκBα degradation via the proteasomal pathway in A7 cells, it had no effect on M2 cells that do not express FLNa (80). However, in our study it is clear that M2 cells consistently respond to 20 ng/ml of TNFα. A difference in the concentration of TNFα used (10 ng/ml in their study *versus* 20 ng/ml in our study) may be the contributing factor to this discrepancy.

TNFα is traditionally known for its anti-tumor activity (81). However, more recent evidences suggest that TNFα also has pro-tumor activities in a cell context-dependent manner (82). Since then many tumors have been reported to express TNF receptor as well as producing TNFα (83). Tumor-derived TNFα can function in an autocrine mode as well as paracrine manners. In the autocrine mode, tumor-derived TNFα can elicit anti-apoptotic signaling cascades as well as stimulating tumor growth, migration, and invasion. When functioning in a paracrine mode, TNFα can promote inflammation, EMT transition, angiogenesis, as well as modulating the host immune system to create an immune microenvironment more favorable for tumor growth. Because TNFα is a pro-inflammatory cytokine, activation of normal immune cells to produce TNFα will further perpetuate this cycle. In multiple animal models, TNFα treatment has been shown to dramatically enhance tumor growth and metastasis (84). Accordingly, inhibition of TNFα using anti-TNFα monoclonal antibodies or TNF receptor fusion protein, such as Etanercept (human TNFR1 extracellular portion and human immunoglobulin G1 (IgG1) Fc) decreased tumor growth and metastasis (85). With regard to human cancers, high levels of released IL-1α is a marker for poor prognosis (86, 87). Our findings that PrP is important for TNFα-mediated NF-κB activation and TNFα production in M2 and BxPC-3 provide a potential mechanistic insight into the roles PrP play in tumorigenesis. However, whether our findings have any clinical relevance will require studying additional human tumor cell lines as well as human tissue biopsies.

## Experimental procedures

### Cell lines, reagents, and antibodies

AsPC-1 and BxPC-3 were purchased from American Type Culture Collection (ATCC). M2 melanoma cell line was kindly provided by Professor, Thomas Stossel, Harvard Medical School. Recombinant human TNFα (number 300-01A), EGF (number AF-100-15), IL-1β (number AF-200–01B) (PeproTech, Rocky Hill, NJ); LPS (number L2630 Sigma); rabbit anti-p-IKKα/β (Ser^176/180^, number 2697), anti-p-SAPK/JNK (Thr^183^/Tyr^185^, number 9251), anti-JNK (number 9252), anti-p-P65 (Ser^536^, number 3033), anti-P65 (number 8242), anti-RIP1 (number 3493), anti-FLAG (number 8146), anti-HA (number 3724); mouse anti-IKKα (number 11930), anti-IKBα (number 4814)(CST, Boston, MA); mouse anti-TRAF2 (Santa Cruz Biotechnology, number SC-136999); Mouse anti-β-actin mAb (number KM9001, Tianjin Sungene Biotech, Tianjin, China) were purchased from the indicated manufacturers. Anti-PrP monoclonal antibodies (4H2, 8B4) were produced and characterized as described (88). All other chemicals used in this paper were purchased from Sigma.

### Construction of plasmids

Mammalian expression plasmids of FLAG-tagged RIP1 and TRAF2, RIP1 and TRAF2 were amplified with the primers listed in Table 3 using the cDNA from M2 cells as the templets. The PCR-amplified target sequences were gel purified and subjected to EcoRI and HindIII or BamHI and HindIII digestion at 37 °C for 1 h, respectively. The digested sequences were further gel purified and ligated into pCMV-3*FLAG backbone, respectively, by standard molecular biology techniques. The plasmids were then sequenced and used for transfection as per the manufacturer's protocol. Mammalian expression plasmids of pCMV-HA-tagged-CYLD were amplified with the primers listed in Table 3. The PCR-amplified target sequences from the M2 cDNA as the template were gel purified and subjected to SalI and NotI digestion at 37 °C for 1 h. The digested sequences were further gel purified and ligated into pCMV-HA backbone by standard molecular biology techniques.

**Table 3:** **Primers used to amplify RIP1, TRAF2, and CYLD from M2 cells**

Gene name	Sequence
RIP	
Sense	5′-GGAATTCGCCACCATGCAACCAGACATGTCCTTGAAT-3′
Antisense	5′-CCCAAGCTTTTAGTTCTGGCTGACGTAAAT-3′
TRAF2	
Sense	5′-CGGGATCCGCCACCATGGCTGCAGCTAGCGTGAC-3′
Antisense	5′-CCCAAGCTTTTAGAGCCCTGTCAGGTCCACAAT-3′
CYLD	
Sense	5′-ACGCGTCGACGATGAGTTCAGGCTTATGGAGC-3′
Antisense	5′-ATAGTTTAGCGGCCGCTTATTTGTACAAACTCATTGT-3′

PrP(1–22)-3*FLAG-PrP(23–253) containing EcoRI and HindIII restriction sites were synthesized by General Biosystems (Anhui, China). The products were then digested with EcoRI and HindIII and cloned into the pcDNA3.1(+) backbone. NF-κB luciferase reporter plasmid was kindly provided by Professor Yanyi Wang (State Key Laboratory of Virology, Wuhan Institute of Virology, Chinese Academy of Sciences).

### Knock-out (knockdown) of PrP in M2 or BxPC-3

To generate PrP knock-out M2 and BxPC-3 cell lines, we used two different CRISPR/Cas systems. The primers for PrP knock-out in M2 and BxPC-3 cells were listed in Table 1. To knock-out *PRNP* in M2 cells, the primers (0.1 μm) were annealed and ligated to PX330 (Addgene, number 42230). To generate *PRNP* null BxPC-3 cells, the primers (0.1 μm) were annealed and ligated to pGL3-U6-sgRNA-PGK-puromycin (Addgene, number 51133). The plasmids were sequenced and transfected into M2 or BxPC-3 cancer cells, respectively. Single clones of M2 or BxPC-3 cells were selected and subjected to DNA sequencing and Western blotting. The sense primer for screening PrP knock-out clones was listed in Table 1.

To knockdown PrP in M2 cells, three pairs of small interfering RNA oligos (listed in Table 4) were generated by the GenePharma (Shanghai, China). 20 nm siRNA was transfected into a well of a 6-well plate using 4 μl of PepMute (catalog number SL100571) siRNA transfection reagent. 24 h later, cells were treated by 20 ng/ml of TNFα for 0, 10, 15, 30, and 40 min. Then the cell lysate were collected and subjected to Western blot analysis.

**Table 4:** **RNA oligos used to knockdown PrP in M2 cells**

Oligo name	Sequence
NC	5′-UUCUCCGAACGUGUCACGU-3′
#1	5′-GACCGUUACUAUCGUGAAA-3′
#2	5′-GCAGAUGUGUAUCACCCAG-3′

### Co-immunoprecipitation (co-IP) and immunoblotting analysis

To identify proteins in M2 cells interacting with PrP, we used the profound co-IP approach according to the manufacturer's protocol. Briefly, 2 × 10^6^ M2 and M2-PrP^−/−^ were seeded in 10-cm Petri dishes overnight. 24 h later cell lysates were prepared in lysis buffer containing 20 mm Tris (pH 7.5), 150 mm NaCl, 1 mm EDTA, 1 mm EGTA, 1% Triton X-100, 2.5 mm sodium pyrophosphate, 1 mm β-glycerolphosphate, 1 mm Na_3_VO_4_, 1 mm PMSF, and EDTA-free protease inhibitor mixture were added just before cell lysis. Co-IP was performed with 100 μg of monoclonal antibody 8B4 or isotype control conjugated to 100 μl of beads (Aminolink number 20381, Thermo Scientific, MA), 400 μl of cell lysate was added to the 8B4-conjugated beads and incubated overnight at 4 °C. The protein complex captured by 8B4 was then washed 6 times with 400 μl of lysis buffer. The precipitates were eluted with 0.1 m glycine (pH 2.5–3.0), 1 m Tris-HCl (pH 9.5) added to neutralize the elusions as described (47). After TCA precipitation, 2× sample buffer was added to the eluted PrP immune complex and boiled at 100 °C for 10 min. The protein complex was then subjected to 10% SDS-PAGE. Specific bands appeared in the 8B4 lane by Coomassie Brilliant Blue staining were subjected to mass spectrometry analysis at the core facility of Wuhan University. To confirm the protein identified by mass spectrometry, the protein co-purified with PrP was subsequently immunoblotted with the indicated antibodies.

To confirm interaction between CYLD and PrP in 293T cells. The FLAG-tagged PrP and HA-tagged CYLD in 1 ml of lysate were captured by 1 μg of mouse anti-FLAG antibodies (number KM8002, Tianjin Sungene Biotech, Tianjin, China), which was incubated in 4 °C for 4 h (the capture beads was protein G). After washing 6 times with 1 ml of lysis buffer, the precipitates were boiled in 2× sample buffer at 100 °C for 10 min. The pellet was then centrifuged at 14,000 × *g* and the supernatant subjected to 10% SDS-PAGE. CYLD co-purified with PrP were detected with the anti-HA tag antibody. To further investigate the interaction of PrP and CYLD upon TNFα treatment, profound co-IP was performed as described before, except the M2 cells were treated with TNFα for 0, 15, 30, and 60 min.

To detect the K63-linkage specific polyubiquitination of RIP1 or TRAF2, 1.5 × 10^6^ M2 or M2-PrP^−/−^ cells were seeded in 10-cm dishes for 24 h. After treatment with TNFα for 30 or 0 min as control, cells were washed twice with ice-cold phosphate-buffered saline (PBS). Cell lysate was collected as described above, 1 μg of RIP1 or TRAF2 antibodies together with 10 μl of protein G beads were added to the 1-ml cell lysate, respectively. The immune complex was then incubated overnight at 4 °C. The beads were washed 6 times with 1 ml of lysis buffer. The pellets were then boiled in 2× sample buffer as described above. Western blotting was performed to analyze the K63-linkage specific polyubiquitination of RIP1 or TRAF2 with K63-linkage specific polyubiquitin antibody (number 5621, CST, Boston, MA).

To confirm that the interaction of PrP and CYLD would protect RIP1 and TRAF2 from CYLD, 1.5 × 10^6^ of HEK293T cells were seeded into 10-cm dishes for 24 h, then pcDNA3.1-PrP or its control backbone pcDNA3.1 together with HA-tagged CYLD and FLAG-tagged RIP1 or TRAF2 were transfected into the HEK293T cells. 24 h later, cells were treated or left untreated with TNFα for 30 min. Then the cell lysate was collected and subjected to mouse anti-FLAG antibody-conjugated protein G beads overnight at 4 °C. The beads were washed 6 times with 1 ml of lysis buffer. The pellets were then boiled in 2× sample buffer as described above. Western blotting was performed to detect if there exists the HA tag signal in the immune complex. Isotype control IgG was applied as control to prove the specificity of the reaction.

### Immunofluorescence staining for confocal microscopy

Tumor cells were cultured in poly-d-lysine-coated glass bottom dishes overnight in an incubator at 37 °C with 95% humidity. Cells were then rinsed 3 times with ice-cold PBS and fixed in 4% paraformaldehyde for 15 min at room temperature. To stain PrP in wild-type M2 and BxPC-3 cells or its corresponding *PRNP* null cells, 4H2 was applied in a concentration of 10 μg/ml. The primary antibody reaction was performed at room temperature for 2 h in PBS containing 0.1% Tween 20 (PBS-T). Bound antibody was then probed with Alexa Fluor 488-nm goat anti-mouse-specific antibody. To detect the co-localization of PrP and CYLD, anti-PrP monoclonal antibody 4H2 or rabbit anti-CYLD (Novus Biologicals, NB110-95574) at 10 μg/ml concentration were applied to the wild-type M2 and *PRNP* null M2 cells for 2 h at room temperature. Bound primary antibodies were detected with Alexa Fluor 555 nm-conjugated goat anti-mouse or Alexa Fluor 488-nm goat anti-rabbit specific antibody (Invitrogen). Isotype control antibodies were applied as negative control. To calculate the co-location index of PrP and CYLD: the fluorescence intensity of PrP was defined as X. Co-location of PrP and CYLD fluorescence intensity was defined as Y. The co-location index was calculated as Y/X.

To stain the nuclei p65 in M2 and M2-PrP^−/−^, the cells were fixed in 4% paraformaldehyde for 15 min at room temperature. Cells were then permeabilized with PBS, 0.5% Triton X-100 for 20 min. p65 was detected with p65-specific antibody at room temperature for 2 h. Bound primary antibody was detected with Alexa Fluor 488 nm-conjugated goat anti-rabbit specific antibody. Nuclei were counterstained with DAPI (number 10236276001, Roche Applied Science, Mannheim, Germany).

### Reporter assays

1 × 10^5^ of the M2 and M2-PrP^−/−^ cells were seeded in each well of a 24-well plate in duplicate. 24 h later, cells were transfected with Lipofectamine 2000 (Invitrogen, REF 11668-019) containing NF-κB luciferase reporter pNiFty (0.002 μg) for each well of a 24-well plate. To normalize the transfection efficiency, pRL-TK (*Renilla* luciferase) reporter plasmid (0.02 μg) was added to each transfection. Luciferase assays were performed using a dual-specific luciferase assay kit (Promega, Madison, WI, number E1960) according to the manufacturer's protocol. Firefly luciferase activities were normalized based on *Renilla* luciferase activities. All reporter assays were repeated at least three times.

### Real-time PCR to quantify TNFα and IL-6

RNA from M2, BxPC-3, and their corresponding *PRNP* null cells treated or untreated with TNFα were isolated using the total RNA purification kit (GeneMark, number TR01-150). 1 μg of RNA was reverse transcribed using a PrimeScript^TM^ RT reagent kit with gDNA eraser (TaKaRa, Shiga, Japan, number RR047A). Aliquots of product were subjected to real-time PCR analysis to quantify the mRNA expression level of tested genes. Each reaction was run in triplicate while the mRNA was diluted 10 times in a final reaction volume of 20 μl. Melting curves were performed to ensure that only a single product was amplified. *GAPDH* was used as a reference gene. Gene-specific primers were listed in Table 5.

**Table 5:** 

Gene name	Sequence
*GAPDH*	
Sense	5′-GACAAGCTTCCCGTTCTCAG-3′
Antisense	5′-GAGTCAACGGATTTGGTCGT-3′
*TNFA*	
Sense	5′-GCCGCATCGCCGTCTCCTAC-3′
Antisense	5′-CCTCAGCCCCCTCTGGGGTC-3′
*IL-6*	
Sense	5′-TTCTCCACAAGCGCCTTCGGTC-3′
Antisense	5′-TCTGTGTGGGGCGGCTACATCT-3′
*P65*	
Sense	5′-GGGGACTACGACCTGAATG-3′
Antisense	5′-GGGCACGATTGTCAAAGAT-3′

Real-Time PCR to quantify the transcription level of p65 in M2 and M2-PrP^−/−^. The mRNA level of p65 from M2 and its corresponding *PRNP* null cells were analyzed using a similar method as that used to quantify *TNFA* and *IL-6* in M2 and BxPC3 cells p65-specific primers were listed in Table 5.

### Subcellular fractionation of p65

1.5 × 10^6^ M2 or M2-PrP^−/−^ cells were seeded in 10-cm dishes 24 h before being harvested. After treatment with TNFα for 30 or 0 min as control, cells were washed twice with ice-cold PBS. The NE-PER Nuclear and Cytoplasmic Extraction Reagents (Thermo, number 78833) were used to extract subcellular fractionation as per the manufacturer's protocol. In brief, 500 μl of ice-cold CER I was added to the cell pellet that contains 10 μl of mixture and 5 μl of PMSF in the tubes. The tubes were vigorously vortexed for 15 min to fully suspend the cell pellet. After a 10-min incubation on ice, 27.5 μl of CER II, which contains 5.5 μl of mixture and 2.75 μl of PMSF were added to the tube and then incubated for 5 min. After that, the tubes were centrifuged at 14,000 × *g*, the supernatant was transferred to the pre-chilled new tubes. The insoluble fraction left in the tubes was solved in ice-cold NER solution after a series of vortex-incubation circles. All these procedures were performed at 4 °C.

### Enzymatic-linked immunosorbent assays

To quantify the medium TNFα: 3 × 10^5^ M2 or M2-PrP^−/−^ cells were seeded in each well of 6-well plates. 24 h later, the cells were left treated or untreated for 3 h by TNFα (20 ng/ml), the supernatant was collected by centrifugation at 1000 × *g* for 3 min to discard the cell debris. Then the secreted TNFα were analyzed using the ELISA kits for human TNFα (BOSTER, number EK0525) as the manufacturer's instructions. Briefly, 100 μl of supernatant was added to each well of pre-coated 96-well plates in duplicate while the same volume of culture medium was used as control. The plate was incubated at 37 °C for 90 min. The culture medium was discarded without washing. Bound antigen was detected with HRP-labeled human TNFα antibody, which was incubated for 60 min and washed thoroughly. After that, the 100 μl of ABC solution was added to plate for 30 min. Then the reaction was detected by 90 μl of 3,3′,5,5′-tetramethylbenzidine substrate. The reaction was stopped by the addition of 100 μl of 3,3′,5,5′-tetramethylbenzidine stop buffer. Absorbance was measured at 450 nm. The experiments were repeated three times.

### Re-introducing PrP into PRNP null M2 cells

pcDNA3.1(+)-PrP(1–22)-3*FLAG-PrP(23–253) or pcDNA3.1 (+) as a negative control were transfected into M2-PrP^−/−^. After selection with G418 (500 μg/ml) for 2 weeks, cells were immediately subjected to treatment with TNFα for different periods of time. The cell lysates were made as above. Separated proteins were blotted with antibodies specific for p-p65, p65, p-JNK, and JNK etc. Bound primary antibodies were further probed with HRP-conjugated secondary antibody.

### Statistical analyses

For the experiments of reporter assays and ELISA, Student's *t* test (two-tailed) was performed to analyze the statistical significance between samples. For the immunofluorescence staining for PrP and CYLD, F-test was performed to analyze their significance of co-location index. Quantitative data are expressed as the mean and mean ± S.E. *, *p* < 0.05 was considered statistically significant. **, *p* < 0.01 was considered statistically very significant.

## Author contributions

G. W. and C. L. designed the experiments; G. W., T. M., and Z. G. performed the experiments; G. W., T. M., Z. G., J. W., M. S, and C. L. analyzed the data; G. W., M. S., and C. L. wrote the paper.

## Supplementary Material

Supplemental Data

## Footnotes

2 The abbreviations used are:

PrP: prion protein
GPI: glycosylphosphatidylinositol
PI-PLC: phosphatidylinositol-specific phospholipase C
CYLD: cylindromatosis
S1P: sphingosine 1-phosphate
co-IP: co-immunoprecipitation
FLNa: filamin A.
