# Supplementary material for: Prion protein is required for tumor necrosis factor α (TNFα)-triggered nuclear factor κB (NF-κB) signaling and cytokine production
Source: J Biol Chem. 2017 Sep 12;292(46):18747–59. doi: 10.1074/jbc.M117.787283 (PMC5704461; doi:10.1074/jbc.M117.787283)
Supplement: Supplemental Data [file supp_292_46_18747__index.html]

Prion protein is required for tumor necrosis factor alpha (TNFα)-triggered nuclear factor kappa B (NF-κB) signaling and cytokine production — Prion protein is required for tumor necrosis factor alpha (TNFα)-triggered nuclear factor kappa B (NF-κB) signaling and cytokine production — Prion protein is required for tumor necrosis factor α (TNFα)-triggered nuclear factor κB (NF-κB) signaling and cytokine production — Prion protein mediates NF-κB signaling — Supplemental Data 

# Prion protein is required for tumor necrosis factor α (TNFα)-triggered nuclear factor κB (NF-κB) signaling and cytokine production

## Supplemental Data

- Supplemental figures and figure legend (.pdf, 1.0 MB) - Supplemental figures and figure legend
