# Supplementary material for: Prion protein is required for tumor necrosis factor α (TNFα)-triggered nuclear factor κB (NF-κB) signaling and cytokine production
Source: J Biol Chem. 2017 Sep 12;292(46):18747–59. doi: 10.1074/jbc.M117.787283 (PMC5704461; doi:10.1074/jbc.M117.787283)
Supplement: Supplemental Data [file 10.1074_M117.787283_jbc.M117.787283-1.pdf]

Prion protein is required for tumor necrosis factor alpha (TNFα)-triggered nuclear factor kappa B (NF-κB) signaling and cytokine production

Gui-Ru Wu[1, 2], Tian-Chen Mu[3], Zhen-Xing Gao[1], Jun Wang[1], Man-Sun Sy[4], Chao-Yang Li[1,5*]

[1] Wuhan Institute of Virology, Chinese Academy of Sciences, State Key Laboratory of Virology, 44 Xiao Hong Shan Zhong Qu, Wuhan, 430071, China
[2] University of Chinese Academy of Sciences, Beijing, 100000, China
[3] Department of Life Sciences, Wuhan University, Wuhan, 430010, China
[4] Department of Pathology, Case Western Reserve University, Cleveland, OH, 44106
[5] Wuhan Brain Hospital, No.5 Huiji Road, Jiang'an District, Wuhan, 430010, China

Running title: prion protein mediates NF-κB signaling

* To whom correspondence should be addressed: Wuhan Institute of Virology, Chinese Academy of Sciences, State Key Laboratory of Virology, 44 Xiao Hong Shan Zhong Qu, Wuhan, 430071, China. Tel: 01186-2787198751, E-mail: cyli@wh.iov.cn

sFigure 1.

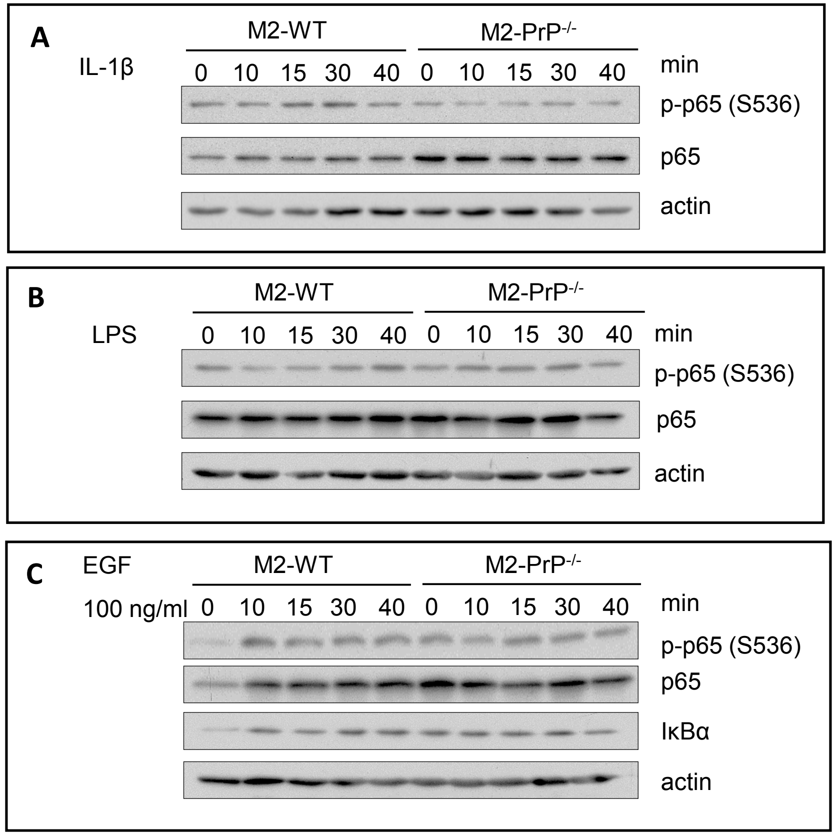

sFigure 1. IL-1β, EGF and LPS do not stimulate the NF-κB signaling in M2 cells. (A) M2 and M2-PrP$^{-/-}$ cells were treated with IL-1β (20 ng/ml) for 0, 10, 15, 30, and 40 minutes. Both of them showed no activation of NF-κB signaling. (B) M2 and M2-PrP$^{-/-}$ cells were treated with LPS (50 ng/ml) for 0, 10, 15, 30, and 40 minutes. Upon induction the M2 and M2-PrP$^{-/-}$ cells showed no activation of NF-κB signaling. (C) M2 and M2-PrP$^{-/-}$ cells were treated with EGF (100 ng/ml) for 0, 10, 15, 30, and 40 minutes. Both of them showed no activation of NF-κB signaling.

sFigure 2.

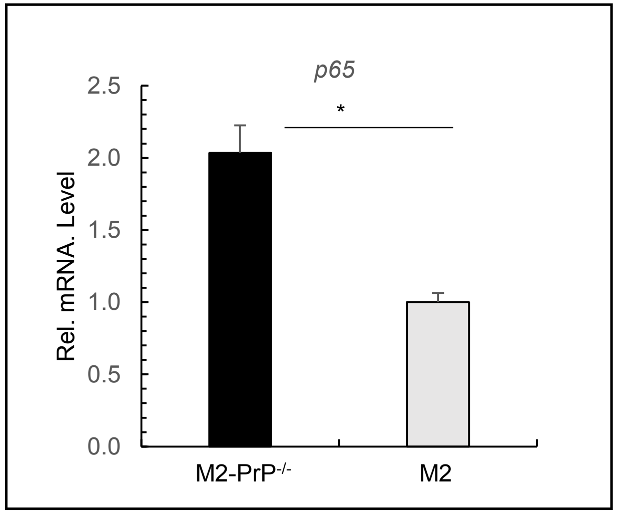

sFigure 2. Loss of PrP in M2 cells potentiates the transcription of p65. Knockout of PrP increases the transcription of p65 in *PRNP* null M2 cells compared to $PrP^{+/+}$ M2 cells.
